# c-Jun Amino-Terminal Kinase is Involved in Valproic Acid-Mediated Neuronal Differentiation of Mouse Embryonic NSCs and Neurite Outgrowth of NSC-Derived Neurons

**DOI:** 10.1007/s11064-016-2167-7

**Published:** 2017-03-21

**Authors:** Lu Lu, Hengxing Zhou, Bin Pan, Xueying Li, Zheng Fu, Jun Liu, Zhongju Shi, Tianci Chu, Zhijian Wei, Guangzhi Ning, Shiqing Feng

**Affiliations:** 1 Department of Orthopaedics, Tianjin Medical University General Hospital, No. 154 Anshan Road, Heping District, Tianjin, 300052 People’s Republic of China; 2 Key Laboratory of Immuno Microenvironment and Disease of the Educational Ministry of China, Department of Immunology, Tianjin Medical University, No. 22 Qixiangtai Road, Heping District, Tianjin, 300070 People’s Republic of China; 3 Kosair Children’s Hospital Research Institute at the Department of Pediatrics, University of Louisville School of Medicine, Louisville, KY 40202 USA

**Keywords:** Neural stem cells, Valproic acid, Neuronal differentiation, Neurite outgrowth, JNK

## Abstract

Valproic acid (VPA), an anticonvulsant and mood-stabilizing drug, can induce neuronal differentiation, promote neurite extension and exert a neuroprotective effect in central nervous system (CNS) injuries; however, comparatively little is known regarding its action on mouse embryonic neural stem cells (NSCs) and the underlying molecular mechanism. Recent studies suggested that c-Jun N-terminal kinase (JNK) is required for neurite outgrowth and neuronal differentiation during neuronal development. In the present study, we cultured mouse embryonic NSCs and treated the cells with 1 mM VPA for up to 7 days. The results indicate that VPA promotes the neuronal differentiation of mouse embryonic NSCs and neurite outgrowth of NSC-derived neurons; moreover, VPA induces the phosphorylation of c-Jun by JNK. In contrast, the specific JNK inhibitor SP600125 decreased the VPA-stimulated increase in neuronal differentiation of mouse embryonic NSCs and neurite outgrowth of NSC-derived neurons. Taken together, these results suggest that VPA promotes neuronal differentiation of mouse embryonic NSCs and neurite outgrowth of NSC-derived neurons. Moreover, JNK activation is involved in the effects of VPA stimulation.

## Introduction

Neural stem cells (NSCs) have the ability to proliferate; self-renew; and differentiate into oligodendrocytes, astrocytes, and neurons; NSCs are also considered a potential therapeutic tool for treating central nervous system (CNS) injuries [1–3]. In NSC-based therapy, neuronal differentiation is a key step in the regeneration and replacement of lost neurons and neural networks [4, 5]. However, NSCs produce only a small number of neurons under normal conditions. Therefore, it is important to explore strategies to promote more efficient neuronal differentiation of NSCs. Moreover, neurite outgrowth is a key cellular aspect of neuronal differentiation and is crucial for neural plasticity and synaptic formation [6].

At present, a multitude of factors have been shown to promote NSC differentiation into neurons, such as neurotrophin-3 (NT-3), NG2, Mash1, bone morphogenetic protein 4, and all-trans retinoic acid (RA) [7–10]; more recent studies reported that valproic acid (VPA), an effective and safe mood stabilizer that has been used in the treatment of bipolar diseases and different epilepsy syndromes for several decades [11, 12]. Recently, the application of VPA has expanded to several clinical trials of treating HIV and various cancers [12–14]. Furthermore, increasing in vitro and in vivo evidence demonstrated that VPA acts as a neurotrophic compound through its ability to increase neurite outgrowth, promote differentiation, protect neurons, and enhance neurogenesis [15–22]. Our previous study has observed that VPA enhanced the neuronal differentiation of embryonic NSCs derived from rat [16], but the relevant mechanisms were not explored. In addition, neuronal differentiation of adult female Fisher 344 rats hippocampal neural progenitor cells (NPCs), embryonic Sprague–Dawley rat-derived NPCs, 46C mouse ESC-derived NPCs, and spinal cord injury (SCI) rat-derived spinal neural stem/precursor cells (NSPCs) were found to be upregulated by VPA [17–20]. Moreover, VPA induced neurite outgrowth in human mesenchymal stem cells (MSCs) and SH-SY5Y neuroblastoma cells [21, 22]. However, the underlying molecular mechanism of VPA-induced neuronal differentiation and neurite outgrowth is complex and remains to be elucidated.

c-Jun N-terminal kinase (JNK), a subfamily of the mitogen-activated protein kinases (MAPKs), has a variety of cellular functions, including cell survival, apoptosis, and programmed cell death [23, 24]. Moreover, accumulating evidence suggests that JNK is involved in embryogenesis, neurite formation, neurite outgrowth, regeneration, neuronal differentiation, and memory formation in the nervous system [25–28]. At present, JNK activation is reported to be implicated in the regulation of neuronal differentiation or neurite outgrowth [29, 30]. Meanwhile, Yamauchi et al. reported that JNK participates in VPA-induced neurite outgrowth of mouse neuroblastoma N1E-115 cells [31]. Therefore, it is reasonable to speculate that JNK activation is required for VPA-induced neuronal differentiation of NSCs and neurite outgrowth of NSC-derived neurons.

In this study, NSCs were obtained from the embryonic mouse cortex; the ability of VPA to enhance their differentiation to neurons, astrocytes, or oligodendrocytes was determined, and neurite outgrowth of mouse NSC-derived neurons was assessed. Furthermore, the requirement of JNK activation in the neurotrophic effect of VPA on the neuronal differentiation of NSCs and neurite outgrowth of NSC-derived neurons was determined.

## Materials and Methods

### Animals

C57BL/6 mice were purchased from the Radiation Study Institute Animal Centre at Tianjin Medical University (Tianjin, China). All experiments were performed in accordance with the Ethics Committee of Tianjin Medical University and complied with the U.S. National Institutes of Health Guide for the Care and Use of Experimental Animals. Every effort was made to minimize the suffering and number of mice used.

### Isolation and Culture of Mouse Embryonic NSCs

NSCs were isolated from mouse embryonic cortex (E13.5) and cultured using the method of Reynolds and Weiss [32, 33], with slight modifications. Briefly, forebrain cortices were cut into small pieces, incubated with accutase (Invitrogen, Carlsbad, CA, USA) and triturated through a 26-gauge needle. The pellets were suspended in growth medium [Dulbecco’s modified Eagle’s medium (DMEM):F-12 medium (DMEM/F12) (Invitrogen) supplemented with epidermal growth factor (EGF, 20 ng/ml), basic fibroblast growth factor (bFGF, 20 ng/ml), leukaemia inhibitory factor (LIF, 10 ng/ml; both from PeproTech, Rocky Hill, NJ, USA), heparin (2.5 μg/ml, Tocris Bioscience, Minneapolis, MN, USA), 2% B27 supplement, l-glutamine (1 mM), penicillin and streptomycin (both 100 U/ml) (all from Invitrogen)]. The cells were incubated in a humidified incubator at 37 °C with 5% CO_2_, and half the medium was exchanged every 2–3 days. After 5–7 days in vitro, the cells proliferated as free-floating neurospheres. Floating neurospheres were collected and passaged after enzymatic dissociation with accutase and mechanically triturated with fire-polished glass pipettes.

### Cell Differentiation and Treatments

For cell differentiation, third-passage neurospheres were dissociated and plated 5.0 × 10^4^/cm^2^ in 12-well cell culture plates coated with poly-l-lysine (PLL, 0.1 mg/ml, Sigma-Aldrich, St. Louis, MO, USA) in differentiation medium [DMEM: F12 medium containing B27 supplement and 1% foetal bovine serum (FBS; Invitrogen)]. Cells were exposed to 1 mM VPA (Sigma-Aldrich) for up to 7 days considering that several lines of evidence have demonstrated that 1 mM is a desirable concentration for the neuronal differentiation of NPCs [16–18]. Furthermore, to investigate the involvement of JNK in response to VPA stimulation, the specific JNK inhibitor SP600125 (Calbiochem, San Diego, CA, USA) was suspended in dimethyl sulfoxide (DMSO; Sigma-Aldrich) and applied to treat NSCs with VPA at 10 μM for 7 days after plating. As SP600125 was resuspended in 0.1% DMSO, an equivalent volume of DMSO was added to control culture medium and VPA-treated NSC culture medium. Therefore, three groups were included: DMSO-treated control culture group, VPA-treated culture group, and VPA plus with SP600125 culture group.

### Immunocytochemistry

Immunocytochemistry was performed as described previously [16]. Cells were washed with PBS, fixed with 4% paraformaldehyde, permeabilized with 0.3% Triton X-100 (Sigma-Aldrich), and blocked with 10% normal goat serum (NGS; Invitrogen). Then, the cells were incubated overnight at 4 °C with the following primary antibodies: rabbit anti-nestin (1:500; Sigma-Aldrich), rabbit anti-β-III-tubulin (1:500; Covance, Princeton, NJ, USA) or rabbit anti-microtubule-associated protein-2 (MAP-2) (1:200; Millipore, Billerica, MA, USA), rabbit anti-glial fibrillary acidic protein (GFAP) (1:1000; Abcam, Cambridge, UK), and rabbit anti-CNPase (1:100; Cell Signaling Technology, Danvers, MA, USA). The cells were then washed with PBS and incubated with Alexa Fluor 555-conjugated goat anti-rabbit (1:500; Invitrogen). 4′6′-diamidino-2-phenylindole (DAPI; Beijing Solarbio Science & Technology Co., Beijing, China) was used to stain nuclei, and fluorescence was detected using a fluorescence microscope (IX71, Olympus, Tokyo, Japan).

### Quantification of Neuronal Differentiation of NSCs and Neurite Outgrowth of NSC-Derived Neurons

**Fig. 1:**
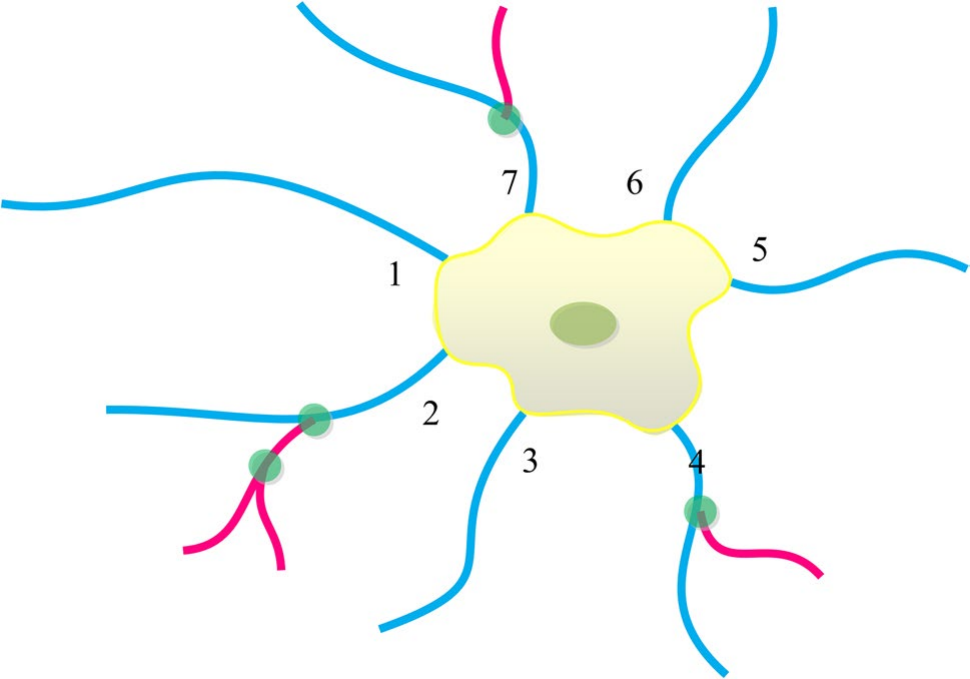
Quantitative assessment of neurite outgrowth. Primary neurites (*blue*) are processes that originate from the cell body and are numbered *1*–*7*; the longest neurite is labelled as number *1*. Branches (*pink*) are processes that originate from a primary neurite. Branch point (*green*) is the point at which a branch originates from a primary neurite or another branch. (Color figure online)

Images were captured from ten randomly selected fields per slide with a 20× objective; 500 cells were counted, and images were analysed using Image Pro plus 6.0 software (Media Cybernetics, Silver Spring, MD, USA). Percentages of differentiated cells were calculated by counting the number of marker-positive cells and total DAPI-stained cells. For neurite assays, the following parameters were measured among NSC-derived β-III-tubulin-positive neurons (as shown in Fig. 1): number of primary neurites and branches, number of neurites, number of branch points, longest neurite length, and total neurite length. A neurite is defined as any process longer than two cell diameters in length [5]; a primary neurite is defined as the neurite that originates from the cell body; a branch is defined as the neurite that originates from a primary neurite [34]; a branch point is defined as the point at which a branch originates from a primary neurite or another branch [34]. To prevent examiner bias, experiments were performed independently and repeated by three persons who were unaware of the others’ research results.

### Western Blot

Western blot analysis was performed as described previously [35], with slight modifications. Briefly, cells were lysed on ice with radioimmunoprecipitation (RIPA) lysis buffer supplemented with protease inhibitor cocktail (Roche, Indianapolis, IN, USA) and sonicated to reduce sample viscosity. Protein concentration was determined using the bicinchoninic acid assay (Pierce, Rockford, IL, USA), equal amounts of protein were separated on 10% SDS-polyacrylamide gels and transferred to polyvinylidene difluoride (PVDF) membranes (Millipore). Western blotting was performed using the following antibodies at 4 °C overnight: rabbit anti-β-III-tubulin (1:5000; Covance); rabbit anti-MAP-2 (1:2000; Millipore); rabbit anti-GFAP (1:5000; Abcam); rabbit anti-CNPase, rabbit anti-JNK, rabbit anti-p-JNK, rabbit anti-c-Jun, rabbit anti-p-c-Jun^ser63^, and rabbit anti-p-c-Jun^ser73^ (all 1:1000; all from Cell Signaling Technology); and mouse anti-β-actin (1:10,000; Sigma-Aldrich). Blots were incubated with horseradish peroxidase-labelled secondary anti-rabbit and anti-mouse antibodies, and immunoreactive bands were visualized on film by enhanced chemiluminescent substrate (Pierce, Rockford, IL, USA) (all 1:10,000, Abcam). Western blots were quantified with ImageJ software from three independent experiments. The intensities of the bands were normalized to β-actin.

### Quantitative Real-Time PCR

The procedure was performed as described previously [36]. Total RNA was extracted from cells with TRIzol reagent (Beijing Solarbio Science & Technology Co), and 1 μg of total RNA was transcribed into cDNA using a first-strand cDNA synthesis kit (Thermo Scientific, USA) according to the manufacturer’s protocol. qRT-PCR was performed on a LightCycler^®^ 96 System (Roche Applied Science, Mannheim, Germany) using a SYBR^®^ Select Master Mix (Applied Biosystems, Foster City, CA, USA). qRT-PCR amplification was performed for 40 cycles using the following thermal profile: denaturation at 95 °C for 15 s, annealing and extension at 60 °C for 1 min. GAPDH was used as an internal control, and fold-changes obtained from three independent experiments were determined using the 2^−ΔΔCT^ method [39]. PCR forward (F) and reverse (R) primers (5′-3′) were as follows: β-III-tubulin (F) CAATGAGGCCTCCTCTCACAA, (R) ATAGTGCCCTTTGGCCCAG; MAP-2 (F) GCCAGCCTCAGAACAAACAG, (R) AAGGTCTTGGGAGGGAAGAAC; GFAP (F) TCAACGTTAAGCTAGCCCTGG, (R) CCCCTTCTTTGGTGCTTTTGC; CNPase (F) TTTACCCGCAAAAGCCACACA; (R) CACCGTGTCCTCATCTTGAAG; BDNF (F) CTGAAGGCGTGCGAGTATTA, (R) AGTCTTTGGTGGCCGATATG; GDNF(F) CAGGGAACTGTGTCTTCCTTAC, (R) CCCTCTAGAAGCAAAGGCTAAA; CDNF (F) GGAGAGAGGATGTGTGCATTAG, (R) CCAACTGTGGGAGGAAGAATAA; and GAPDH (F) AGGTCGGTGTGAACGGATTTG, (R) GTAGACCATGTAGTTGAGGTCA.

### Statistical Analysis

All experiments were reproduced in triplicate. All values were presented as the mean ± standard error of the mean (SEM). Quantitative data were analysed using GraphPad Prism 5.0 (GraphPad Software Inc., San Diego, CA, USA), with Student’s *t* test, χ^2^ test or one-way analysis of variance (ANOVA) and the Bonferroni post hoc test. Statistical significance was set at p < 0.05.

## Results

### NSCs can Proliferate and Differentiate into Different Types of Neural Cells

**Fig. 2:**
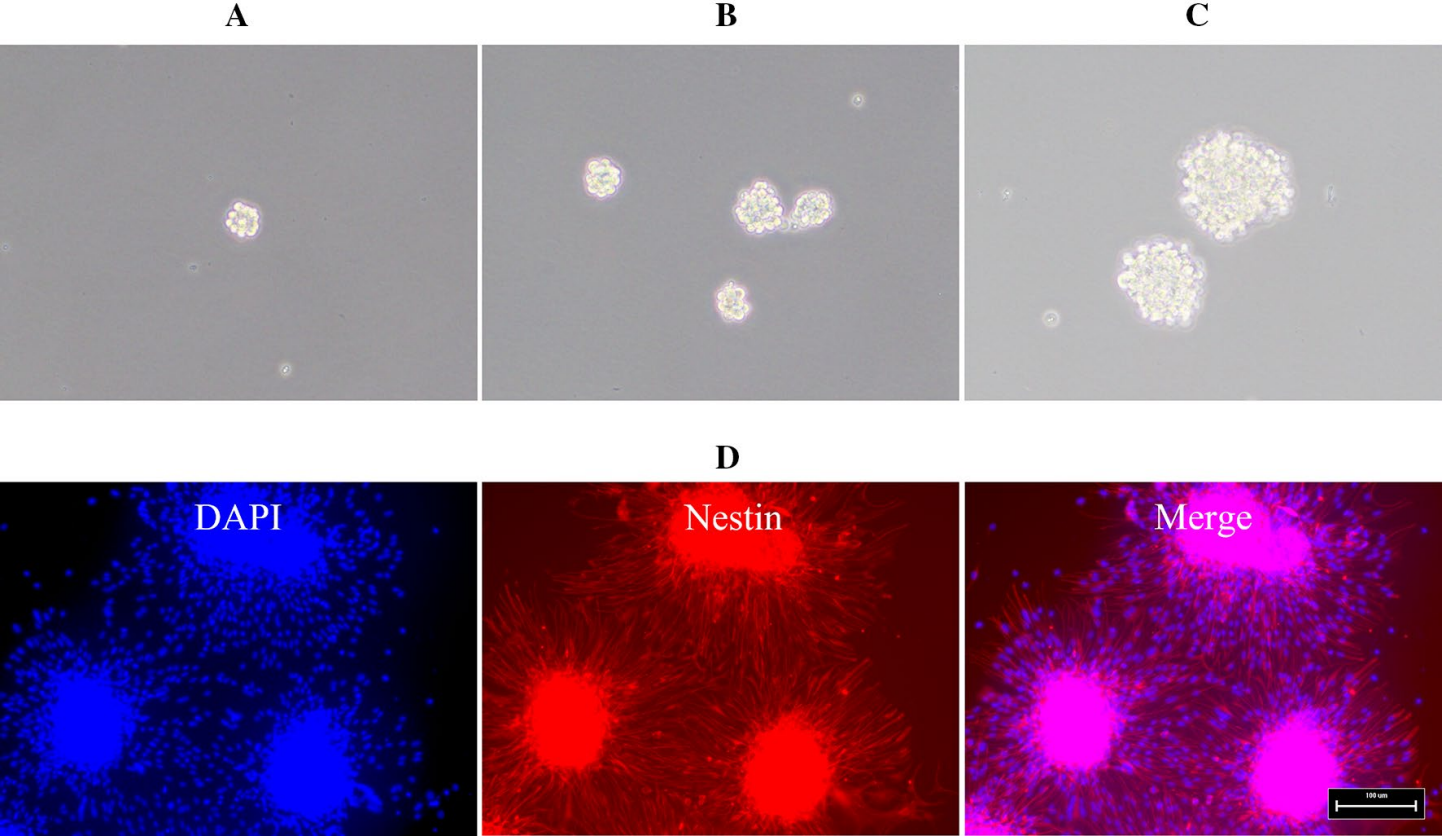
The growth and identification of mouse embryonic NSCs. Mouse embryonic NSCs cultured in growth medium proliferated and formed neurospheres on the second day after primary culture. **a** Phase contrast micrographs of 1-day culture of mouse NSC neurospheres. **b** Phase contrast micrographs of 3-day culture of mouse NSC neurospheres. **c** Phase contrast micrographs of 5-day culture of mouse NSC neurospheres. **d** Mouse embryonic NSCs in neurospheres were stained by immunocytochemistry for nestin. *Scale bar* 100 μm

**Fig. 3:**
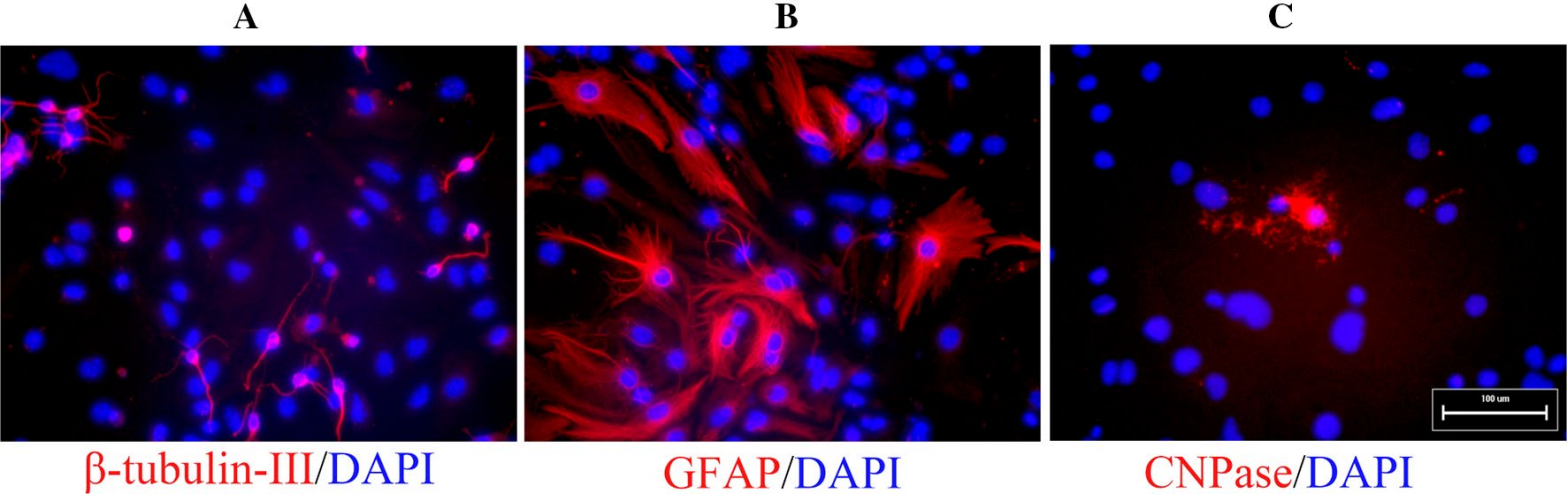
Mouse embryonic NSCs differentiate into different types of neural cells. NSCs were seeded onto PLL-coated plates, cultured in differentiation medium up to 7 days, and immunostained for β-tubulin-III (**a**), GFAP (**b**), and CNPase (**c**). *Scale bar* 100 μm

We isolated NSCs from the brains of E13.5 mouse embryos. On the second day after primary culture, cells cultured in growth medium proliferated and formed neurospheres (Fig. 2a), and neurospheres continued to proliferate when maintained in growth medium (Fig. 2b, c). All of the neurospheres showed strong immunoreactivity to nestin (Fig. 2d). To differentiate NSCs, third-passage neurospheres were dissociated, transferred to coverslips coated with PLL, and then cultured in differentiation medium for up to 7 days; these cells differentiated into β-III-tubulin-positive neurons (Fig. 3a), GFAP-positive astrocytes (Fig. 3b), and CNPase-positive oligodendrocytes (Fig. 3c). These results confirm that we successfully isolated NSCs that differentiated into neurons, astrocytes and oligodendrocytes.

### VPA Treatment Mediated Improved Neuronal Differentiation of Mouse Embryonic NSCs

**Fig. 4:**
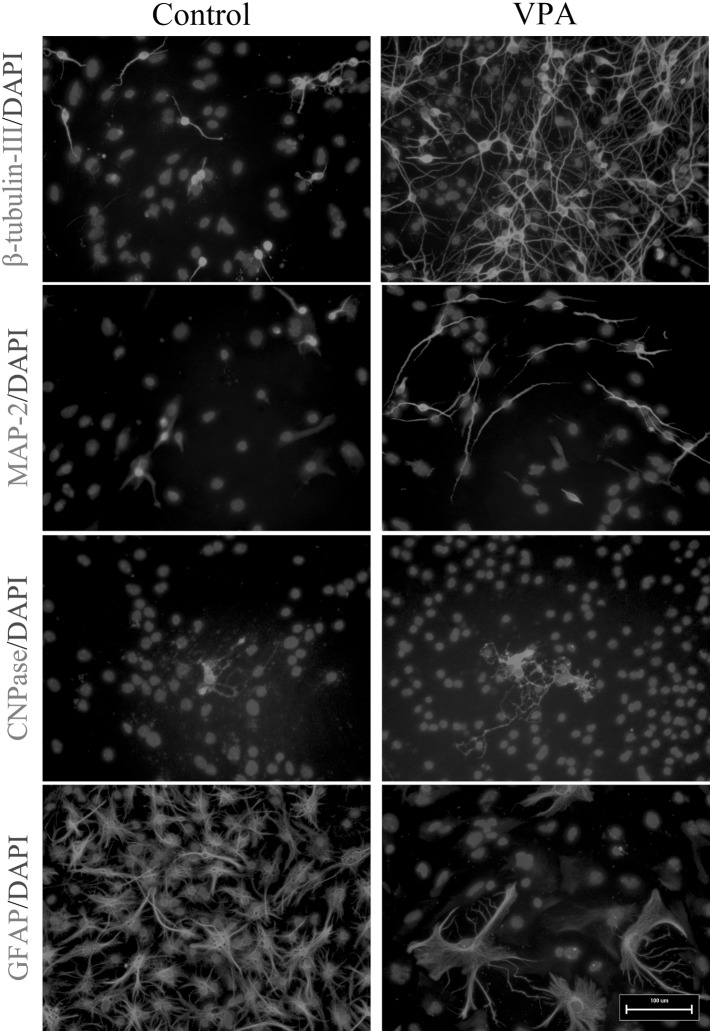
Mouse embryonic NSCs were treated with 1 mM VPA for up to 7 days and immunostained with anti-β-tubulin-III, anti-MAP-2, anti-CNPase, and anti-GFAP. *Scale bar* 100 μm

**Fig. 5:**
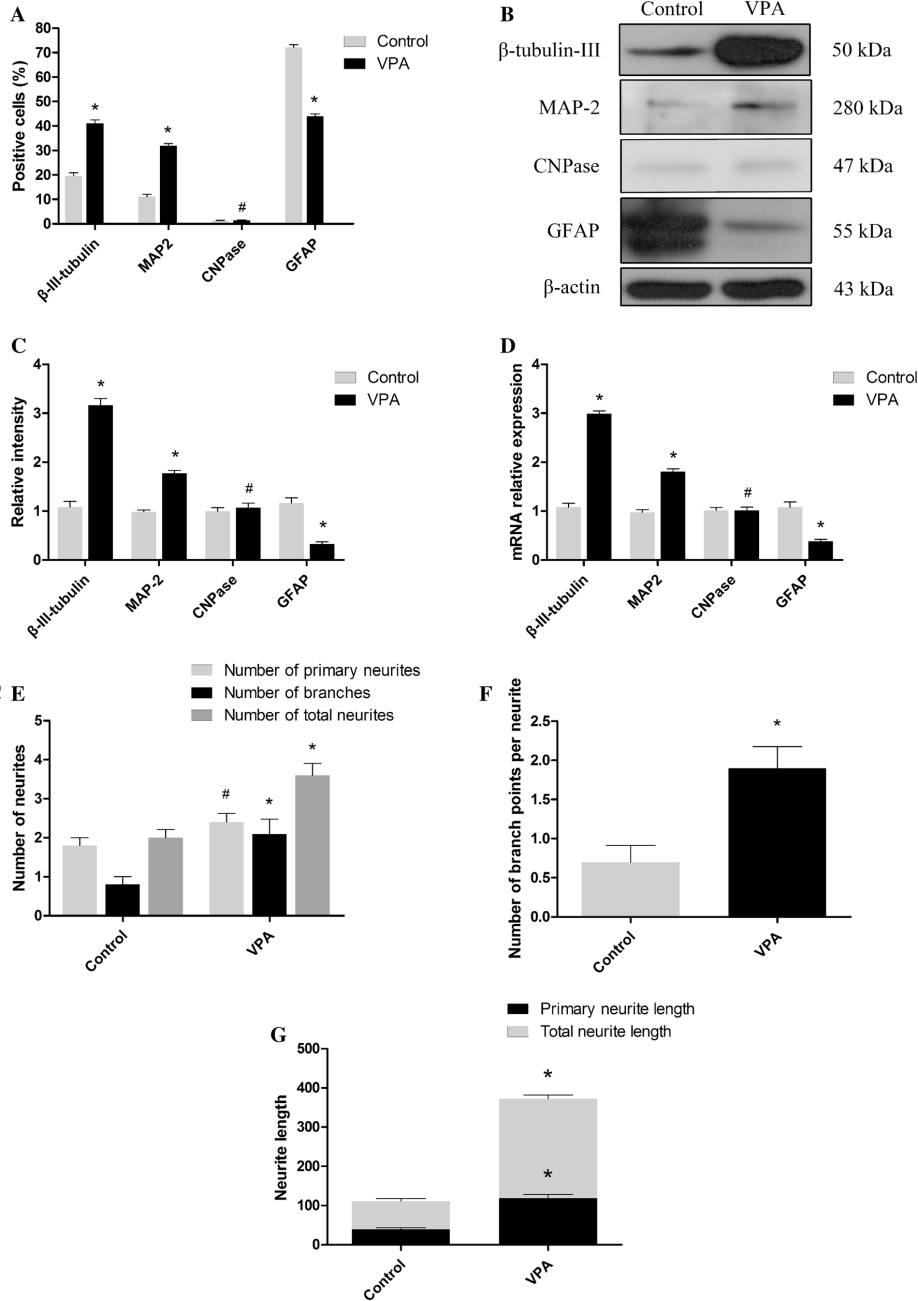
VPA promotes the neuronal differentiation of mouse embryonic NSCs and the neurite outgrowth of NSC-derived neurons. **a** Summary of proportions of mouse embryonic NSC-derived β-tubulin-III-, MAP-2-, CNPase-positive cells, and GFAP-positive cells. **b** Western blot analysis of β-tubulin-III and GFAP. β-actin protein levels were used as a loading control. **c** Summary of the quantification of β-tubulin-III, MAP-2, CNPase, and GFAP levels. **d** Summary of the quantification of β-tubulin-III, MAP-2, CNPase, and GFAP gene expression. **e** Quantification of the number of primary neurites and branches and the number of neurites. **f** Quantification of the number of branch points. **g** Quantification of the longest neurite length and total neurite length. *p < 0.05 compared with the DMSO-treated control cultures; ^#^p > 0.05 compared with the DMSO-treated control cultures

To evaluate whether VPA might influence the capacity of mouse NSCs to differentiate into neurons, mouse NSCs were dissociated into single cells, cultured in differentiation medium and treated with 1 mM VPA for 7 days. In the VPA-treated cultures, 41.20 ± 1.19% of all cells were β-III-tubulin-positive (Figs. 4, 5a), exceeding that observed in DMSO-treated control cultures (18.80 ± 1.08%, p < 0.01) (Figs. 4, 5a; χ^2^ test, p < 0.05). Similarly, a significant increase in the VPA-treated cultures (31.00 ± 1.08 vs 11.60 ± 1.06%) was obtained using MAP-2 antibodies (Figs. 4, 5a; χ^2^ test, p < 0.05). The number of astrocytes decreased in the VPA-treated cultures (43.80 ± 1.05%) compared with the DMSO-treated control cultures (72.90 ± 1.06%) (Figs. 4, 5a; χ^2^ test, p < 0.05). Moreover, the number of oligodendrocytes was not affected by VPA (Figs. 4, 5a; χ^2^ test, p > 0.05).

Furthermore, western blot and qRT-PCR were conducted to confirm the results of the immunocytochemical analyses. The protein and gene expression levels of β-III-tubulin and MAP-2 increased significantly in VPA-treated cultures compared with DMSO-treated control cultures (Fig. 5b–d, t test, p < 0.05), whereas the protein and gene expression levels of GFAP decreased significantly in VPA-treated cultures compared with DMSO-treated control cultures (Fig. 5b–d, t test, p < 0.05). Moreover, the protein and gene expression levels of CNPase remained unchanged (Fig. 5b–d, t test, p > 0.05). Collectively, these data demonstrate that VPA treatment promoted mouse NSCs to predominantly differentiate into neurons.

### VPA Treatment Mediated improved Neurite Outgrowth in Mouse Embryonic NSC-Derived Neurons

Neurite outgrowth was assessed on day 7 in VPA-induced NSC-derived neurons immunostained with β-III-tubulin, and relevant parameters were measured as Fig. 1 shown. The results showed that the neurite outgrowth of VPA-treated NSC-derived neurons was significantly increased compared with DMSO-treated control cultures, including number of branches (Fig. 5e; 2.10 ± 0.384 vs. 0.80 ± 0.20; *t* test, p < 0.05), number of neurites (Fig. 5e; 3.60 ± 0.31 vs. 2.00 ± 0.21; *t* test, p < 0.05), number of branch points (Fig. 5f; 1.90 ± 0.28 vs. 0.70 ± 0.21; *t* test, p < 0.05), longest neurite length (Fig. 5g; 71.89 ± 6.42 vs. 39.51 ± 3.86 μm; *t* test, p < 0.05) and total neurite length (Fig. 5g; 253.43 ± 9.53 vs. 118.85 ± 9.48 μm; *t* test, p < 0.05), whereas no difference was observed in the number of primary neurites (Fig. 5e; 2.4 ± 0.22 vs. 1.80 ± 0.20; *t* test, p > 0.05). Collectively, these data demonstrate that VPA treatment promoted the neurite outgrowth of NSC-derived neurons.

### JNK Activation was Involved in the VPA-Mediated Improvement in Neuronal Differentiation of NSCs and Neurite Outgrowth of NSC-Derived Neurons

**Fig. 6:**
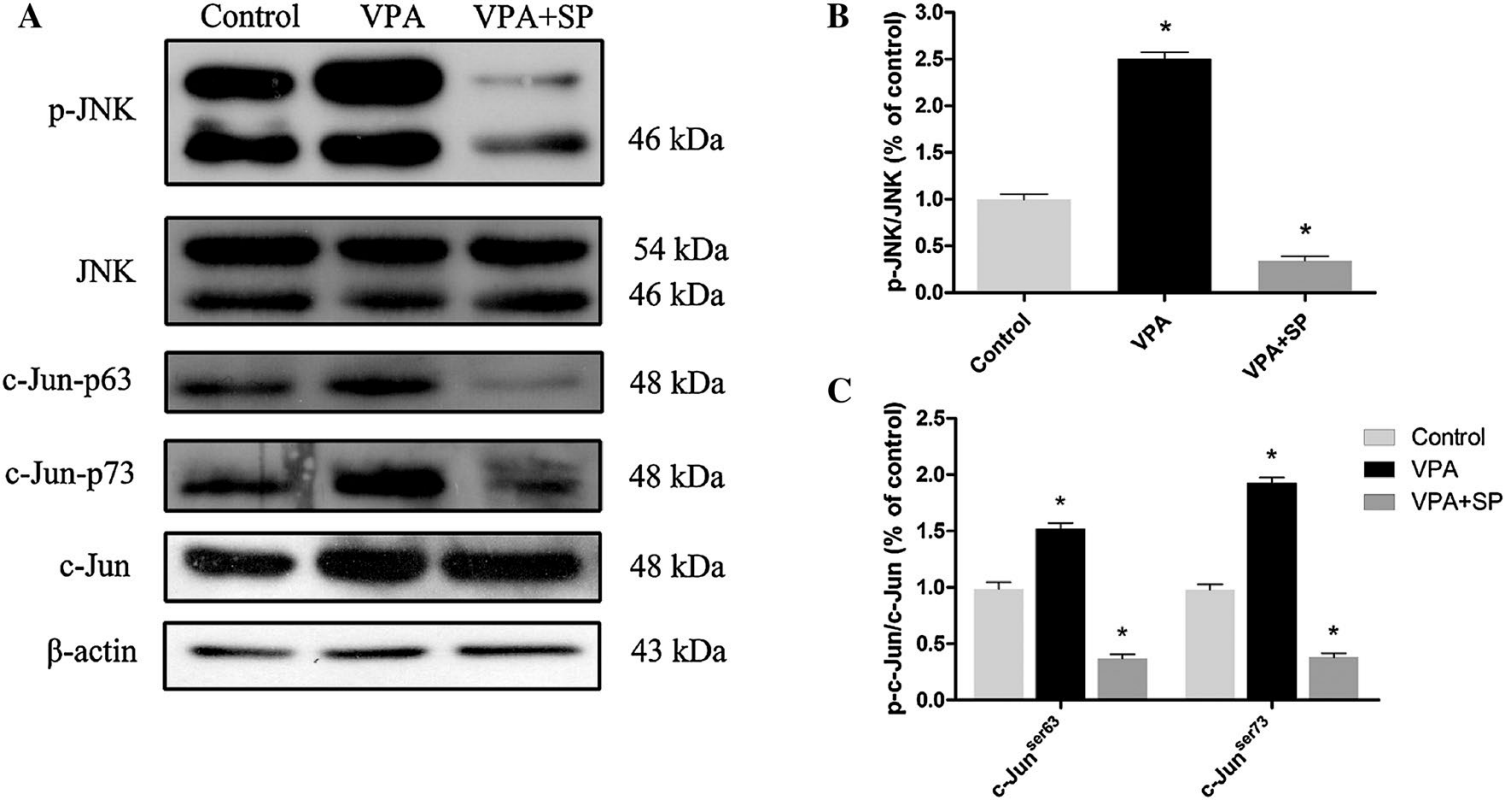
Effect of VPA on of p-JNK, t-JNK, p-c-Jun^ser63^, p-c-Jun^ser73^ and t-c-Jun protein levels. **a** Western blot analysis of p-JNK, t-JNK, p-c-Jun^ser63^, p-c-Jun^ser73^ and t-c-Jun. β-actin protein levels were used as a loading control. **b** Summary of the quantification of p-JNK/t-JNK protein levels. **c** Summary of the quantification of p-c-Jun^ser63^/t-c-Jun and p-c-Jun^ser73^/t-c-Jun protein levels. ^*^p < 0.05 compared with the DMSO-treated control cultures

First, the expression of JNK and c-Jun was detected. As shown in Fig. 6, by day 7, the phosphorylation of JNK-c-Jun was enhanced (one-way ANOVA followed by Bonferroni post-test, both p < 0.05), whereas no change in the total JNK-c-Jun protein levels (one-way ANOVA, p > 0.05) was observed over the VPA-induced neuronal differentiation process. This result indicates that JNK may be an essential component in the control of the neuronal differentiation of NSCs and neurite outgrowth of NSC-derived neurons.

**Fig. 7:**
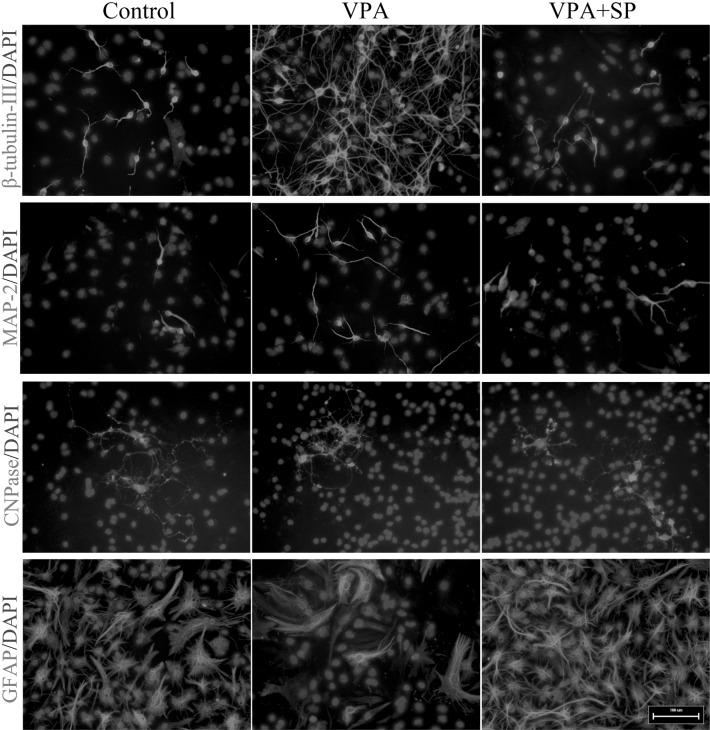
Mouse embryonic NSCs were treated with 1 mM VPA for up to 7 days in the absence or presence of 10 µM SP600125 and immunostained with anti-β-tubulin-III, anti-MAP-2, anti-CNPase, and anti-GFAP. *Scale bar* 100 μm

**Fig. 8:**
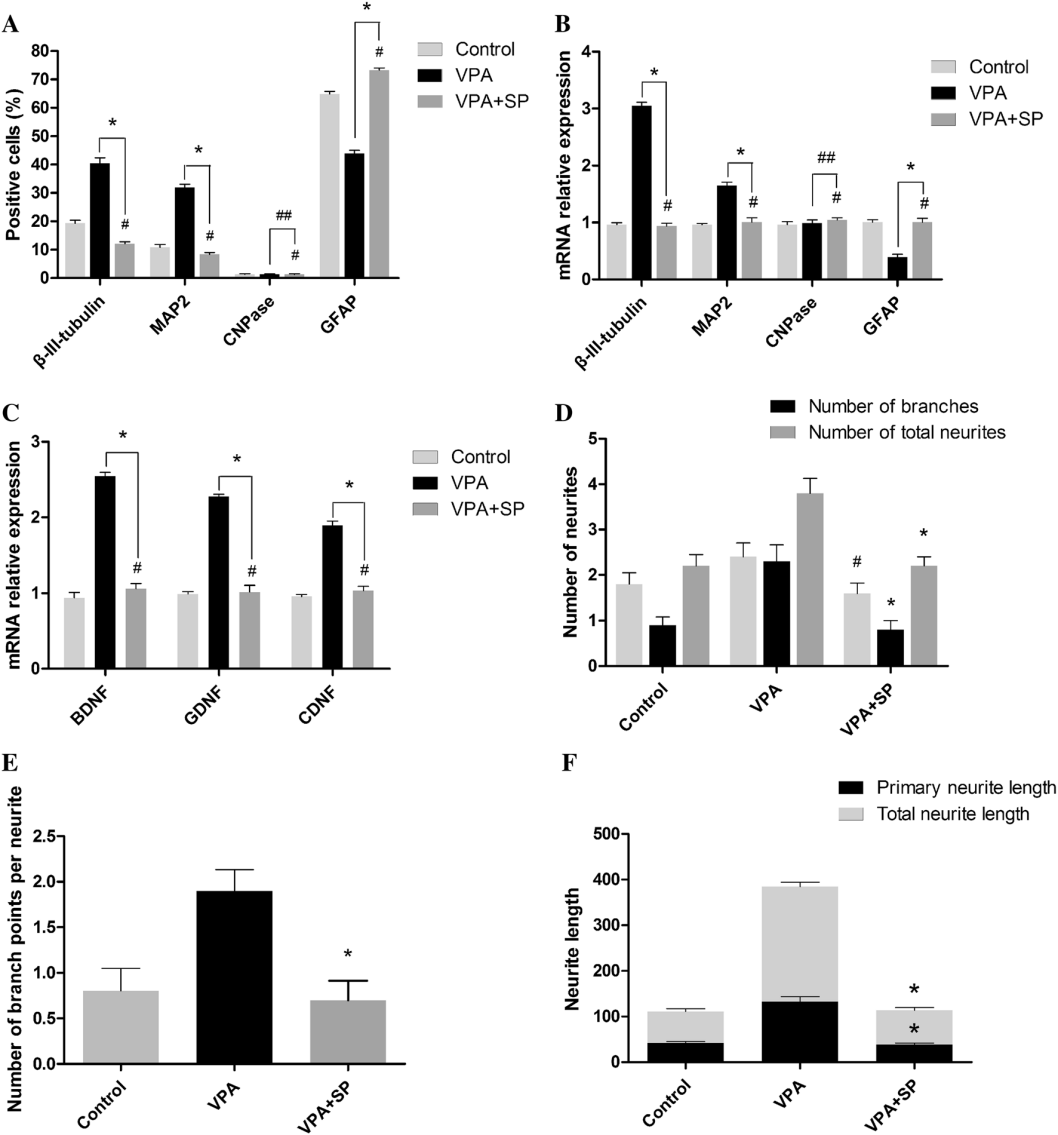
SP600125 decreased the VPA-mediated increase in the neuronal differentiation of mouse embryonic NSCs and the neurite outgrowth of NSC-derived neurons. **a** Summary of the proportions of mouse embryonic NSCs-derived β-tubulin-III-, MAP-2-, CNPase-positive cells, and GFAP-positive cells. **b** Summary of the quantification of β-tubulin-III, MAP-2, CNPase, and GFAP gene expression. **c** Summary of the quantification of BDNF, GDNF and CDNF gene expression. **d** Quantification of the number of primary neurites and branches and the number of neurites. **e** Quantification of the number of branch points. **f** Quantification of longest neurite length and total neurite length. *p < 0.05 compared with the VPA-treated control cultures; ^#^p > 0.05 compared with the VPA-treated control cultures; ^##^p > 0.05 compared with the VPA-treated control cultures

Furthermore, to investigate the involvement of JNK in VPA-mediated neuronal differentiation of NSCs and neurite outgrowth of NSC-derived neurons, SP600125 (10 μM), a selective JNK inhibitor, was applied to block phosphorylation of JNK. As shown in Fig. 6, SP600125 resulted in a significant decrease in phosphorylated JNK-c-Jun (one-way ANOVA followed by the Bonferroni post-test, both p < 0.05). Moreover, the VPA-mediated increased proportions of β-III-tubulin- and MAP-2-positive cells were largely reduced in the presence of SP600125, whereas the percentage of GFAP-positive cells increased (Figs. 7, 8a; χ^2^ test, all p < 0.05). Similar results were obtained from qRT-PCR: β-III-tubulin and MAP-2 gene expression decreased, whereas GFAP gene expression increased, CNPase gene expression remained unchanged (Fig. 8b; one-way ANOVA followed by the Bonferroni post-test, all p < 0.05). Moreover, gene expression levels of BDNF, GDNF, and CDNF increased significantly in VPA-treated cultures compared with DMSO-treated control cultures, whereas they all decreased significantly in the presence of SP600125 (Fig. 8c; one-way ANOVA followed by the Bonferroni post-test, all p < 0.05).

Moreover, immunocytochemical analysis of β-III-tubulin demonstrated that SP600125 reduced the VPA-mediated increase in neurite outgrowth, including number of branches, number of neurites, number of branch points, longest neurite length, and total neurite length of NSC-derived neurons (Fig. 8c–e; one-way ANOVA followed by the Bonferroni post-test, all p < 0.05).

Together, these results implied that JNK activation plays a crucial role in the VPA-induced neuronal differentiation of mouse embryonic NSCs and the neurite outgrowth of NSC-derived neurons.

## Discussion

Here, we successfully obtained NSCs from the embryonic mouse forebrain cortex, and we demonstrated that VPA treatment enhanced neuronal differentiation of mouse embryonic NSCs and neurite outgrowth of NSC-derived neurons over differentiation conditions. Particularly, we showed that JNK activation participates in these processes.

CNS injuries are mainly caused by the progressive death or damage of neurons [19, 37, 38]. NSC transplantation can replace damaged or lost neurons via the ability of NSCs to differentiate into neurons [3, 37, 39]. Therefore, NSCs are excellent candidates for stem cell-based therapies for CNS injuries [2–4]. In NSC-based therapy, neuronal differentiation is an important step in the regeneration and replacement of neurons and neural networks [33]. In addition, neurite outgrowth, a key cellular aspect of neuronal differentiation, is important for neural plasticity and synaptic formation [6]. Based on these findings, we investigated whether VPA can increase the neuronal differentiation of mouse embryonic NSCs and neurite outgrowth of NSC-derived neurons.

VPA, an anticonvulsant and mood stabilizer, is safe and effective and has broad pharmacological effects, such as inducing apoptosis, inhibiting proliferation, and increasing tumour cell immunogenicity [12–14]. Importantly, VPA exerts more divergent and complex actions in the nervous system, where it upregulates neurotrophic factors, such as brain-derived neurotrophic factor (BDNF) and glial cell line-derived neurotrophic factor (GDNF), in astrocytes [40–42]; VPA treatment enhances the neuronal differentiation of haematopoietic stem cells, bone marrow-derived MSCs, sympathoadrenal progenitor cells, adult female Fisher 344 rats hippocampal NPCs, embryonic Sprague–Dawley rat-derived NPCs, 46C mouse ESC-derived NPCs, adult spinal NSPCs from SCI rats, and rat embryonic NSCs [16–21, 43–45]. Moreover, VPA increased the neurite outgrowth of SH-SY5Y neuroblastoma cells, human hippocampal cultures, and MSCs [21, 22, 46]. Additionally, Abematsu and colleagues recently found that VPA administration significantly enhanced the differentiation of transplanted NSCs into neurons in SCI mice [47]. Therefore, we hypothesized that VPA can improve the neuronal differentiation of mouse embryonic NSCs and neurite outgrowth of NSC-derived neurons. Indeed, our study demonstrated that VPA increased the percentage of β-III-tubulin- or MAP-2-positive neurons. However, a previous study has found that VPA-treated mice (300 mg/kg) have a decreased level of postnatal neurogenesis in the hippocampus, this is presumably due to a slower differentiation of the residual NPCs caused by depletion of the NPC pool [48]. These seemingly contradictory findings could imply that VPA-mediated various effects might depend on the cell type, context of VPA usage, and experimental design used. Meanwhile, the effects of VPA on neurite length of NSC-derived neurons were assessed, including number of branches, number of neurites, number of branch points, longest neurite length, and total neurite length. Number of branches and number of neurites are semi-quantitative parameters which do not result in a calibrated measure of neurite length; while number of branch points, longest neurite length, and total neurite length are quantitative parameters which provide a calibrated measure for some aspect of neurite length [34]. Therefore, our experiments successfully confirmed that VPA increased the neurite length of NSC-derived neurons. Moreover, coincident with the idea that CDNF and GDNF mRNA expressions in C17.2 neural stem cells were upregulated by 0.5 or 1.0 mM VPA [40], our results further confirmed that 1.0 mM VPA increased BDNF, GDNF and CDNF level significantly. In addition, a serum VPA concentration of 0.3–1 mM is commonly used for the long-term treatment of epilepsy or mood disorders [12, 46], and several lines of evidence have demonstrated that 1 mM is a desirable concentration for the neuronal differentiation of NPCs [16–18]. Therefore, in this experiment, we chose 1 mM VPA to treat mouse embryonic NSCs. However, the molecular mechanism by which VPA exerts its effects in mouse embryonic NSCs needs to be clarified.

JNK plays an important role in the neuronal differentiation or neurite outgrowth of several cell lines, including dopaminergic neurons, PC12 cells, P19 embryonal carcinoma cells, human neuroblastoma SH-SY5Y cells, and N1E-115 neuroblastoma cells [30, 31, 49–51]. JNK can be activated by a variety of physical and chemical stresses, such as osmotic stress, redox stress, heat shock, or UV irradiation [23, 24]; moreover, neurotrophic factors and cytokines can stimulate the JNK signalling cascade. JNK phosphorylation is required for the NGF-mediated neuronal differentiation of PC12 cells [26], the IFN-γ-mediated neuronal differentiation of NPCs [52], and the RA-induced differentiation of MN9D dopaminergic neuronal cells, as well as the spontaneous neurite outgrowth of dopaminergic neurons [30]. Meanwhile, VPA acts as a neurotrophic growth factor and exerts a variety of neurotrophic effects by enhancing neurogenesis, protecting neurons, and promoting differentiation [21, 44, 46]. In addition, a recent study demonstrated that VPA induces the neurite outgrowth of mouse neuroblastoma N1E-115 cells through a JNK-paxillin unit [31]. Together, these findings raise the possibility that JNK activation is required in the VPA-mediated neuronal differentiation of NSCs and neurite outgrowth of NSC-derived neurons. To verify this hypothesis, we applied the JNK inhibitor SP600125, which is an efficient and specific inhibitor of the JNK that can significantly inhibit the activation/phosphorylation of JNK [53, 54]. In the present study, we showed that the increase in β-III-tubulin- and MAP-2-positive cells mediated by VPA decreased significantly in the presence of SP600125. This result is consistent with the majority of previous findings showing that SP600125 can decrease the proportions of β-III-tubulin-positive cells [17, 18]. Meanwhile, in our study, we showed that SP600125 decreased the increased neurite outgrowth of NSC-derived neurons stimulated by VPA, which is consistent with previous studies demonstrating that SP600125 nearly abolished neurite outgrowth in cultured ES cell-derived neurons and N1E-115 cells [5, 31, 55]. Moreover, the increased BDNF, GDNF and CDNF level regulated by VPA decreased significantly in the presence of SP600125. Collectively, these results imply that JNK activation is involved in the VPA-mediated neuronal differentiation of NSCs and neurite outgrowth of NSC-derived neurons.

The JNK family consists of JNK1, JNK2, and JNK3. JNK1 and JNK2, which are widely expressed in a variety of tissues, are involved in the regulation of neurite outgrowth in spiral ganglion neurons, dorsal root ganglion neurons and PC12 cells [29, 56]; whereas JNK3, which is highly expressed in CNS neurons, the heart, and the testis [57, 58], contributes to the maturation of ES cell-derived cortical neuronal cells and controls the neurite outgrowth of midbrain dopaminergic neurons [59]. Clearly, we suspect these three isoforms to have differential effects on the neuronal differentiation of NSCs and neurite outgrowth of NSC-derived neurons; therefore, further studies should investigate the mechanism of each JNK isoform in the neuronal differentiation of NSCs and neurite outgrowth of mouse NSC-derived neurons. Moreover, several previous studies have demonstrated that HDAC, GSK-3, and Wnt are involved in the VPA-mediated neuronal differentiation of NPCs [45, 60]; it will be of interest to explore these mediators in VPA-mediated neuronal differentiation and neurite outgrowth of mouse NSC in a future study.

In summary, this study demonstrates that VPA improves the neuronal differentiation of mouse NSCs and neurite outgrowth of NSC-derived neurons; moreover, JNK activation was involved in the effects of VPA stimulation.
